# Physician-suggested strategies for deimplementing low-value care in Swedish primary care: a qualitative analysis across system levels

**DOI:** 10.1186/s43058-026-00947-6

**Published:** 2026-04-18

**Authors:** Hanna Augustsson Öfverström, Sara Ingvarsson, Emma Hedberg Rundgren, Marta Roczniewska, Henna Hasson, Per Nilsen, Ulrica von Thiele Schwarz

**Affiliations:** 1 Procome Research Group, Medical Management Centre, Department of Learning, Informatics, Management and Ethics, Karolinska Institutet, Stockholm, Sweden; 2 Center for Epidemiology and Community Medicine, Region Stockholm, Stockholm, Sweden; 3 Centre for Psychiatry Research, Department of Clinical Neuroscience, Karolinska Institutet, Stockholm, Sweden; 4 Department of Health, Medicine and Caring Sciences, Division of Health and Society, Linköping University, Linköping, Sweden; 5 School of Health, Care and Social Welfare, Mälardalen University, Västerås, Sweden

**Keywords:** Primary healthcare, Low-value care, Deimplementation, Implementation strategies, Expert recommendation for implementing change, IGLOO framework

## Abstract

**Background:**

Low-value care (LVC) remains a persistent problem in primary healthcare. While various implementation strategies have been proposed and tested for deimplementation, there is limited knowledge about whether there are strategies uniquely suited to deimplementation. Furthermore, there is limited knowledge about system-level responsibilities for deimplementation. Given that implementation strategies are recommended to be tailored to the specific contextual factors surrounding LVC practices, involving primary care physicians in identifying contextually appropriate strategies holds promise. This study aimed to explore the strategies that primary care physicians suggest could facilitate the deimplementation of LVC and map them to the system levels responsible for initiating and deploying these strategies.

**Methods:**

This qualitative study was based on responses to an open-ended survey question asking physicians to suggest strategies that could facilitate the deimplementation of LVC practices within primary healthcare in Sweden. A national sample of 441 primary care physicians responded. Responses were analyzed via deductive content analysis categorized by the Expert Recommendations for Implementing Change (ERIC) compilation. Responses that could not be categorized via ERIC were analyzed inductively. The identified strategies were then mapped to the system levels (individual, group, leader, organization and overarching system) responsible for deploying these strategies.

**Results:**

Strategies were identified across all nine original ERIC domains, representing 39 (53%) of the original ERIC strategies, and later additions for deimplementation. Additionally, a set of strategies categorized under a new domain, develop better evidence, was identified. Most strategies were suitable for deployment at the organizational (31 strategies) and overarching (31 strategies) levels. Sixteen strategies were best suited for the group level, thirteen for the leadership level, and two for the individual level.

**Conclusion:**

Primary care physicians proposed strategies across all nine ERIC domains, with additional strategies beyond those previously described, including develop better evidence. From the physicians’ perspective, deimplementation cannot rely solely on individuals but requires coordinated action across the organizational and system levels. Broader structural, policy, and cultural changes would support physicians and ensure that responsibility for deimplementation is shared across the healthcare system.

**Supplementary Information:**

The online version contains supplementary material available at 10.1186/s43058-026-00947-6.

Contributions to the literature

- This study systematically applies and extends the Expert Recommendations for Implementing Change (ERIC) compilation to deimplementation, identifying strategies across all existing domains and introducing a new domain, “Develop better evidence.”
- This finding shows that most suggested implementation strategies require action at the organizational and overarching levels, challenging the dominant focus on individual providers.
- It highlights several underexplored but potentially powerful strategy areas, such as stakeholder collaboration, infrastructure reform and patient engagement, that have received little prior empirical attention.
- It contributes to developing contextually grounded, physician-informed strategies for deimplementation that reflect the realities of primary care practice.

## Introduction

The use of low-value care (LVC) remains a persistent challenge in healthcare, leading to negative consequences for both individual patients and the healthcare system as a whole [1, 2]. LVC is defined as “services that provide little or no benefit to patients, have potential to cause harm, incur unnecessary cost to patients, or waste limited healthcare resources” [3]. Examples of LVC practices within primary care include unnecessary diagnostic tests, nonindicated imaging, and nonindicated medications [4]. It is challenging to fully quantify the prevalence of LVC [5], as the measurement methods varies and prevalence of LVC differs across countries, healthcare providers and practices [6–8]. In a study among primary care physicians across five European countries, over 50% of the physicians perceived LVC to be a problem in their own practice, and over 80% perceived it to be a problem in their countries’ healthcare system [9].

Historically, greater emphasis has been placed on processes for implementing evidence-based interventions rather than on reducing or eliminating LVC. However, there is a growing recognition of the need to use structured and planned processes to reduce or stop the use of LVC, i.e., deimplementation [10]. Guidelines, recommendations and lists of practices to reduce or discontinue are becoming increasingly common. The most well-known example is the international initiative Choosing Wisely [11], in which more than 500 recommendations of practices that should be reduced have been developed. However, identifying only practices that should not be used or where use should be limited is not sufficient to deimplement LVC [12].

Various strategies to deimplement LVC have been proposed and, to some extent, have also been tested. A scoping review examined deimplementation strategies and compared them to the implementation strategies outlined in the Expert Recommendations for Implementing Change (ERIC) compilation [13]. Of the 73 strategies in the ERIC compilation, 36 (49%) were used for deimplementation. Education for professionals, audits and feedback, and reminders were the most used strategies. However, several gaps in the literature concerning deimplementation strategies exist. First, it is unclear whether the ERIC implementation strategies not yet used for deimplementation are inherently unsuitable or have simply not been tested [13]. This gap may mean that potentially effective strategies to reduce LVC have been overlooked. Second, the review identified deimplementation strategies not included in ERIC, suggesting that there may be room for additional strategies that are uniquely tailored to deimplement LVC. Third, similar to implementation strategies, deimplementation strategies need to be selected on the basis of an assessment of local barriers and facilitators for the use of LVC [13–15]. However, a systematic review of randomized controlled trials showed that only 18% of the reported deimplementation strategies have been tailored to context-specific factors [15]. Similarly, a scoping review of deimplementation strategies found a very limited use of tailoring [13]. The lack of tailoring may be one reason for the mixed evidence for the effectiveness of deimplementation strategies [14, 16, 17].

Barriers to and facilitators of implementation exist across multiple levels of the healthcare system [18, 19]. These include *individual professional levels*, such as knowledge, attitudes and habits; *individual patient levels,* such as expectations, preferences and demands; *group or organizational levels,* such as work processes, infrastructure and organizational culture; and *outer context levels*, such as policies, reimbursement models and regulatory frameworks. Despite this multilevel complexity of LVC, the focus of deimplementation strategies is often on individual healthcare professionals. Even when there is a need to address individual-level barriers, such as a lack of knowledge addressed through some form of education, this narrow focus overlooks the fact that decisions to deploy such strategies are typically made at higher levels. For example, primary care physicians report that their use of LVC is often shaped by contextual factors, many of which lie beyond their control [4, 9, 20]. At the same time, they often feel solely responsible for these decisions, suggesting limited system-level support for deimplementation [20].

While ERIC provides a taxonomy of strategy types, it does not specify the system level at which responsibility for initiating or deploying a strategy resides. To provide insight on where in the system responsibility for action lies, an additional multilevel analytical lens is required. Whereas we have found no framework developed specifically to categorize de-implementation strategies by system level, several models emphasize the importance of addressing multiple, interacting levels to optimize change in complex settings such as healthcare, e.g., Mcleroy’s Social Ecological Model [21], Sociotechnical systems [22], Ferlie & Shortells four levels of change [23] and the Rainbow model for integrated care [24]. These models consistently underscore interdependence across different parts of a system, such as individuals, teams, leadership structures, organization, and the wider policy and regulatory context, to drive change. Although the terminology and number of levels vary between frameworks, they span from the individual (I) and group or team (G) levels to the leader (L), organizational (O), and overarching or societal context (O) levels. These are summarized in the IGLOO framework [25, 26]. By offering a straightforward representation of system levels, IGLOO facilitates systematic mapping of strategies to the levels at which they are initiated and deployed. Such a level-specific perspective makes visible the distribution of resources, decision authority, and responsibilities across the system, and supports alignment between identified barriers and the actors best positioned to address them.

Primary care plays a central role in addressing the negative consequences of LVC because of its position as the first point of contact, with a large proportion of the population visiting primary care every year and its position as a gatekeeper for secondary care [4]. However, it has also been argued that the literature on LVC overlooks the specific local conditions that exist in primary care [27]. By investigating the perspective of primary care physicians who have profound knowledge about the context in which care takes place, strategies that physicians perceive as helpful and support deimplementation can be identified. This includes strategies that may not have been previously described and tested in the deimplementation literature.

This study aimed to explore the strategies that primary care physicians suggest could facilitate the deimplementation of LVC and map them to the system levels responsible for initiating and deploying these strategies.

## Materials and methods

### Design

The study had a qualitative design based on an open-ended question in a cross-sectional national survey. The study is reported according to the Standards for Reporting Qualitative Research (SRQR) [28] (Additional file 1).

### Setting

The study was conducted in a national sample of primary care physicians representing both public and private primary care centers (PCCs). In Sweden, primary care is organized and delivered by 21 regions. Both public and private (approx. 47%) service providers who have a care agreement with the region deliver primary care. Providers are funded and governed according to the same rules but can differ in how they are organized and managed. Primary care physicians are typically employed, not independent contractors, with the provider fee operating at the PCC level, which does not affect individual physicians [29]. Most physicians working in primary care in Sweden are specialists in general/family medicine [30].

In Sweden, do-not-do recommendations are published on the National Board of Health and Welfare´s national guidelines [31]. Choosing Wisely was launched in 2024 [32], after the data collection for this study was completed, but the international Choosing Wisely initiative was already known to some of the respondents and some were aware that the Swedish Society of Medicine was working on developing a Swedish Choosing Wisely.

### Participants

Primary care physicians were recruited during the winter and spring of 2023. The online survey was disseminated through advertisements in a newsletter from the national organization for specialists in general medicine and through a Facebook group for physicians working in general practice in Sweden that has approximately 4500 members. Recruitment was supported by a paid social media advertisement targeting primary care physicians. In total, 593 individuals responded to the survey, of which 441 (74%) provided responses to the open-ended questions that were analyzed in this study. Of these, 296 (67%) were female, 140 (32%) were male, and three did not disclose their gender. Nationally, 58% of primary care physicians in 2021 were women [30]. Most of the participants were 30%–39% years (37%) or 40%–49% years (36%), 16% were aged 50%–59% years, 9% were aged 18%–29% years, and 2% were over 60% years. Years of work experience in their current occupation varied between less than one year (8%), 1–2 years (14%), 3–5 years (27%), 6–10 years (22%) and more than 10 years (29%). All 21 healthcare regions in Sweden were represented, and most of the physicians worked in public PCCs (62%), 35% worked in private PCCs, and 3% worked in both public and private PCCs. This distribution is in line with the national pattern, where 64% of primary care physicians were employed in public PCCs in 2021 [30].

### Data collection

The invitation to participate included a link to a webpage that contained information about the purpose of the survey, participants’ rights and information about data management, as well as contact information for the researchers. After reading this information, individuals provided informed consent to participate and accessed the online survey through a link.

The survey included background questions about the respondents (gender, age, work experience) and the workplace (geographical region and service provider ownership (public or private)). The following open-ended question was posed: What type of intervention do you believe would facilitate the deimplementation of ineffective practices within healthcare?

The survey also included vignettes, i.e., different scenarios concerning the provision of LVC, and the results have been reported previously [33].

### Analysis

Data in the form of text in response to the open-ended question were cleaned and organized. All nonempty responses were screened, and entries such as “no ideas” or “I don’t know” were removed. A total of 441 responses remained for analysis. When a response included more than one proposed strategy, it was separated into separate rows, resulting in 749 individual suggestions. When the exact same strategy was written by several participants (e.g., more professional training), we kept it once for sorting (all other instances were deleted), leaving 691 suggestions. All the suggestions were transferred to Miro, a digital collaboration platform [34] where they were analyzed by six of the researchers (HAÖ, SI, EHR, MR, HH, UvTS) using qualitative abductive content analysis [35]. The analysis began with a deductive phase followed by an inductive phase. Qualitative content analysis was chosen because it allows for both manifest and latent interpretation of the data. An abductive approach was used to enable a more comprehensive understanding of the material, as it made it possible to identify strategies not previously included in the ERIC compilation.

The Expert Recommendations for Implementing Change (ERIC) compilation [36] was used as a framework for deductive coding. ERIC compiles 73 discrete implementation strategies that have been clustered into nine domains of strategies [37]. Perry et al. [38] suggested an additional strategy: assess and redesign workflow. Ingvarsson et al. [13] applied ERIC for deimplementation and proposed four additional types of deimplementation strategies: policy and regulations, black box warnings, communication tools, and accountability tools. Thereafter, Kien et al. [16] added another strategy relevant for deimplementation, called changes in the scope and nature of benefits and services. We used the original ERIC strategies and the additional strategies [13, 16, 36, 38] to categorize the strategy suggestions. Suggestions that did not fit within any of the domains or strategies were inductively analyzed. The open-ended responses varied in depth and breadth and ranged from describing strategies needed for de-implementation in a few words to richer descriptions encompassing several sentences. Therefore, categorization required interpretation of the meaning of the suggestions. The analysis was conducted in analysis meetings where all six researchers assessed all suggestions and agreed on the interpretation and categorization of them. When uncertainty arose regarding the appropriate categorization, all six researchers discussed the categorization until a consensus was reached. Thereafter, the data for each category was synthesized by HÖ and reviewed by all researchers. Examples of the coding via ERIC can be found in Additional file 2.

Because our research question explicitly concerned not only which strategies physicians suggest but also where in the system responsibility for action lies, an additional multilevel analytical lens was required. In a second step, four of the researchers (HAÖ, UvTS, EHR, and SI) applied the IGLOO framework [25, 26] to match the strategies to the system level where the decision to initiate the strategy can be made. The IGLOO framework offers a parsimonious representation of interacting system levels (individual, group, leadership, organizational, and overarching/social) and allows systematic mapping of strategies to loci of decision authority. Applying IGLOO enabled us to examine the distribution of responsibility for deimplementation across the healthcare system.

We defined the individual level as the individual physician; the group level as the PCC; the leader level as PCC management; the organizational level as the primary care organization within a healthcare region; and the overarching/social context as the wider overarching system, encompassing primary care across multiple regions, secondary care, government agencies and national educational institutions.

Matching was performed by considering the decision latitude of key stakeholders (i.e., what an individual or group can realistically decide to implement) and the efficiency of implementation (e.g., while an individual physician could develop training materials, it is often more resource efficient to do so at the group or organizational level). Not all strategy suggestions included a manifest description of the appropriate level for strategy deployment. Many participants described processes, actors, or decision pathways without explicitly naming the system level, but their accounts implicitly indicated where authority or responsibility resided. In these cases, latent interpretation was necessary. The latent interpretation of the appropriate level was grounded in participants’ descriptions and in the researchers extensive knowledge and experience of the structural features of the Swedish healthcare system. See additional file 2 for examples of manifest and latent interpretation of IGLOO levels. One category of strategies can be categorized at several levels. For example, participants’ suggestions of *local consensus discussions* were categorized at both the group and leadership levels, as they were described by the participants as suitable to be initiated from either. We deliberately distinguished this from the level where the strategy is experienced or enacted, which may differ. For example, a clinical decision support system is typically created at the organizational level but is used by individual physicians. We chose to categorize strategies by decision level to better reflect how responsibility and authority for initiating and supporting deimplementation efforts are distributed across the system. This approach provides more actionable insights for deimplementation.

All researchers involved in analyzing the data have profound knowledge about deimplementation strategies. Four of the six researchers who conducted the first part of the analysis have extensive experience using the ERIC compilation. One of the researchers had expertise in using the IGLOO framework. Owing to the nature of the data collection method, none of the researchers had any direct contact or personal relationships with the participants.

## Results

The physicians suggested strategies across all ERIC domains, and the later made additions [13, 16, 38], representing 39 (53%) of the original individual ERIC strategies and four out of the five later added strategies for deimplementation [13, 16]. Additionally, a new domain was identified: *develop better evidence*, including four strategies. Additionally, a few new strategies within existing domains were identified. These were: *appropriate level of care* within the Change infrastructure domain and two new strategies: *aligned directives across the health system level*; *develop national guidelines* that reflect policy and regulation, a previously identified strategy [13], which we labeled a domain here.

The suggested strategies span multiple levels, with deployment possible at the individual (I), group (G), leader (L), organizational (O), or overarching/social (OS) levels (IGLOO), or across several levels simultaneously. Most of the strategies were considered suitable for deployment at the organizational (31 strategies) and overarching/social (31 strategies) levels. Sixteen strategies were best suited for the group level, thirteen for leadership, and two for the individual level. Table 1 presents the identified deimplementation strategies categorized by ERIC domains and additions [13, 16, 38], with the addition of novel strategies not covered by existing ERIC strategies. The table also specifies the most appropriate system level responsible for deploying each strategy.

**Table 1:** Suggested strategies organized by domains and strategies and their level of deployment according to the IGLOO model

Domain	Suggested strategies	Deployment levels				
		**I**	**G**	**L**	**O**	**OS**
**Use evaluative and iterative strategies**	Audit and provide feedback		G	L	O	S
	Conduct local needs assessment		G	L		
	Conduct cyclical small tests of change		G	L		
	Develop and organize quality monitoring systems		G	L	O	OS
	Accountability tool [13]				O	
		**-**	**4**	**4**	**3**	**2**
**Provide interactive assistance**	Facilitation		G	L		
	Provide clinical supervision	I	G	L		
		**1**	**2**	**2**	**-**	**-**
**Adapt and tailor to context**	Tailor strategies		G	L	O	OS
	Promote adaptability					OS
	Use data warehousing techniques					OS
		**-**	**1**	**1**	**1**	**3**
**Develop stakeholder interrelationships**	Conduct local consensus discussion		G	L		
	Inform local opinion leaders				O	
	Build a coalition				O	OS
	Promote network weaving					OS
	Visit other sites				O	
	Use advisory boards and workgroups					OS
	Involve executive boards				O	OS
		**-**	**1**	**1**	**4**	**4**
**Train and educate stakeholders**	Conduct educational meetings		G	L	O	
	Conduct ongoing training					OS
	Make training dynamic		G			
	Create a learning collaborative				O	
	Develop educational materials					OS
	Distribute educational materials				O	OS
	Work with educational institutions					OS
		**-**	**2**	**1**	**3**	**4**
**Support clinicians**	Remind clinicians		G		O	
	Revise professional roles			L	O	OS
	Facilitate relay of clinical data to providers					OS
		**-**	**1**	**1**	**2**	**2**
**Engage consumers**	Decrease demand					OS
	Use mass media					OS
	Prepare patients to be active participants	I				
	Communication tool [13]		G			OS
		**1**	**1**	**-**	**-**	**3**
**Utilize financial strategies**	Alter incentive/allowance structures			L	O	OS
	Fund and contract for the clinical innovation				O	OS
	Develop disincentives				O	
	Use capitated payments				O	
	Use other payment schemes				O	OS
		**-**	**-**	**1**	**5**	**3**
**Change infrastructure**	Mandate change including priorities			L	O	
	Change physical structure and equipment		G		O	
	Create or change credentialing and/or licensure standards				O	OS
	Change accreditation or membership requirements				O	OS
	Change liability laws					OS
	Assess and redesign workflow [38]		G	L	O	
	Appropriate level of care (new)				O	OS
		**-**	**2**	**2**	**6**	**4**
**Policy and regulations **[13]	Aligned directives across health system levels (new)		G		O	OS
	Develop national guidelines (new)				O	OS
		**-**	**-**	**-**	**2**	**2**
**Changes in scope and nature of benefits and services **[16]	Changes in scope and nature of benefits and services				O	OS
		-	-	-	1	1
**Develop better evidence** (new)	Include time and cost in guidelines				O	OS
	Applicable guidelines				O	OS
	Clarify the evidence for recommendations				O	OS
	R&D for practices with unclear evidence		G		O	
		**-**	**1**	**-**	**4**	**3**
**Total sum of strategies**		**2**	**16**	**13**	**31**	**31**

^*^IGLOO stands for Individual (I), which was operationalized as the individual physician; Group (G), which was operationalized as a PCC; Leader (L), which was operationalized as the management of a PCC; Organization (O), which was operationalized as the organization of primary care within a healthcare region; and Overarching/social (OS), which was operationalized as the wider healthcare system, including primary health care in several regions, secondary care, government agencies and educational institutions at the national level

### Use evaluative and iterative strategies.

The physicians suggested assessing and delivering feedback on LVC use (*audit and provide feedback*). This strategy may be deployed across system levels, except by individual physicians. Audit and feedback could promote team discussions and individual reflection on one’s own LVC use (G) and support guideline adherence discussions in yearly performance management dialog (L). The use of benchmarking across PCCs (O, OS) was further suggested.

“Ongoing monitoring from the region, for example through the statistics that are presented, where my health centre is compared with other centres in the same area. If we then see that we issue 50% more referrals for colonoscopies, that will likely make us stop and reflect.” (Audit and provide feedback)

Physicians also request systems for quality monitoring and follow-up to track LVC use and associated costs at local, regional, or national levels (G, L, O, OS) (*develop and organize quality monitoring systems*). The development of quality indicators focused on effective care may help reduce LVC. Assessing the prevalence of LVC and identifying deimplementation needs at the PCC level (*conduct local needs assessment*) (G, L) was also proposed. Projects should be piloted locally via small cycles of change, with successful initiatives scaled to other PCCs (*conduct cyclical small tests of change)* (G, L).

To increase accountability, physicians suggested requiring justification for LVC tests/referrals, e.g., by requiring a justification in the electronic medical record (*accountability tool*). Another proposal was to allow referral recipients, such as imaging departments, to reject referrals with reference to LVC (O).

### Provide interactive assistance

Physicians emphasized the importance of addressing LVC in medical student training (*provide clinical supervision*). This could involve supervisors being present during care appointments to help students critically assess the need for diagnostic tests and referrals (I). Physicians have suggested using external facilitation to support collaborative discussions aimed at creating a supportive climate for reducing LVC (*facilitation*) (G, L).

“Sufficient allocated time for debriefing immediately after the visit for trainee doctors would likely reduce overdiagnosis by allowing them to feel confident and deliberate about postponing further investigation for the moment.” (Provide clinical supervision)

### Adapt and tailor to context

Physicians reported that LVC may result from overly detailed guidelines, standardized care processes, and excessive top-down control. Guidelines were sometimes perceived as poorly suited to the realities of primary care. To address this, framing guidelines as supportive tools rather than rigid rules, clarifying when exceptions are appropriate, and allowing physicians greater discretion to adapt care to individual patients (*promote adaptability*) (OS) have been suggested.

Another suggested strategy was to improve data sharing across care providers (*use data warehousing techniques*) (OS), including public, private, and online services. This was seen as a way to prevent patients from seeking LVC repeatedly from different providers until one agreed to deliver it. *Tailor strategies* were the only strategy in this domain that extended beyond the system level. It included examples at the individual (I), group (G), and organizational (O) levels, indicating that tailoring may need to occur across multiple levels.

“The choice of intervention depends on the specific situation and the underlying causes of using ineffective methods. It is probably most effective to use a combination of these strategies to achieve the best result.” (Tailor strategies)

### Develop stakeholder interrelationships

Physicians have proposed strategies to strengthen collaboration and dialog across different levels of the healthcare system. They emphasized the importance of regular meetings at the PCC to discuss LVC (*conduct local consensus discussions*) (G). These meetings could focus on evaluating evidence, sharing experiences with difficult patient cases, and exploring ways to deny patient requests for LVC. Leadership was seen as essential for fostering a climate that supports such discussions (L).

“Physicians at the same workplace should follow the guidelines in the same way to reduce patients going around to different colleagues… if it’s a difficult patient with strong demands, then it should be discussed at a physician meeting and a joint decision given to the patient.” (Conduct local consensus meeting)

Physicians suggested increasing dialog between primary and secondary care and between different PCCs (*build* *a coalition)* (O, OS). Visiting secondary care units to exchange knowledge (*visit other sites*) (O) and creating networks focused on topics such as overdiagnosis (*network weaving*) (OS) were proposed to support cross-professional collaboration.

Trusted individuals, such as local opinion leaders, were seen as influential in encouraging the abandonment of entrenched practices (*inform local opinion leaders)* (O). Physicians also called for greater involvement of healthcare professionals in decisions about care delivery and administration and for improved communication between professionals and senior management (*use advisory boards and workgroups*) (OS). Finally, physicians highlighted the role of political actors in shaping patient expectations and demand. They suggested that executive boards should be involved in recognizing and addressing how political promises may unintentionally promote LVC (*involve executive boards*) (O, OS).

### Train and educate stakeholders

Physicians have suggested training initiatives to support the deimplementation of LVC. The most frequently mentioned strategy was to *conduct educational meetings* at the PCCs (G). Suggested examples included sessions on the definition and implications of LVC, Choosing Wisely, guideline updates, evidence appraisal, and health economy. Interactive formats were emphasized (*make training dynamic*) (G), including realistic case examples and group discussions. Other suggested strategies included timely dissemination of new evidence via email, newsletters and seminars (*distribute educational material*) (O). Targeted training for specific groups, such as temporary staff, and continuous joint training for primary and secondary care providers were also proposed (*create a learning collaborative)* (O).

“Discussions led by the medical director at the local workplace. Based on guidelines and facts about which investigations/treatments have a high time needed to treat, and which are common ‘time thieves’ or ‘money drains’ in our unit.” (Conduct educational meetings)

Physicians also requested ongoing training (*conduct ongoing training*) and increased time for professional development (OS). Additionally, integrating LVC-related content into medical education (*work* *with educational institutions*) (OS) and providing clear, accessible information about LVC relevant to primary care (*develop educational material)* (OS) were suggested. Physicians further requested written patient information for use during consultations and on national health information platforms (OS).

### Support clinicians

Strategies to support clinicians include various types of reminders to aid decision making concerning LVC (*remind clinicians*) (G, O). The physicians emphasized the need for fewer but more effective decision support systems, preferably integrated into electronic medical records to help them navigate among guidelines at the point of care (O). Additional suggestions included providing cost information (G) and implementing mechanisms to deny nonrecommended test orders. Analog reminders such as articles in professional journals or newsletters (OS) were also proposed.

The physicians also emphasized the need to reduce administrative burden and allow more time for clinical work and patient interaction (*revise professional role*s) (L, O, OS). Strengthening the societal role of primary care physicians was considered important for enhancing patients’ trust in their expertise. Increased access to relevant clinical data was suggested to support care planning, including avoiding LVC (f*acilitate relay of clinical data to providers*) (OS).

“Fewer and simpler decision-support tools that also take cost‑effectiveness and displacement effects into account.” (Remind clinicians)

### Engage consumers

Strategies to engage consumers ranged from those deployable by individual physicians, such as involving patients in care decisions (*preparing patients to be active participants)* (I), to strategies the physicians requested from the broader system, which targeted the general population (OS). Physicians have suggested that public education on risks with overdiagnosis and overtreatment and prioritization of necessary care could *decrease demand* for LVC (*increase* *demand in ERIC*). The proposed communication strategies included national campaigns (e.g., choosing wisely), informational displays in waiting rooms, and targeted social media posts. Mass media could be a viable information channel to increase awareness about LVC and mitigate health anxiety and lobbyism, such as pushing for tests (*use of mass media*).

“For example, conducting informational campaigns in public places such as: ‘Did you know that a cough caused by a common cold resolves on its own without cough medicine and is usually caused by a virus that does not require antibiotic treatment?’ Today, however, we tend to hear messages emphasizing that it should be easy to contact a doctor, which I interpret as meaning that people are encouraged to seek medical care for all sorts of issues, as if everything were equally important, low-value care versus complex multimorbidity. “ (Decrease demand)

The physicians also emphasized the need for support for how to communicate with patients about LVC (*communication tool*). They perceived it as challenging to deny patient requests for LVC, particularly under time pressure. The suggested strategies included training in patient communication and the provision of clear arguments for avoiding certain LVC practices (G). Readily available patient information about LVC was also proposed (OS).

### Utilize financial strategies

The physicians proposed financial strategies primarily targeting organizational and overarching levels, reflecting the limited influence of individual or group-level actors over financial policy. One exception was the strategy *alter incentive/allowance structures*, which was identified as having the potential to reduce LVC by modifying quality indicators that promote unnecessary care. This strategy also included suggestions for how managers could adjust communication around incentives to better align with evidence-based practices (L, O, OS).

“Link reimbursement to the use of effective methods and the results they generate.” (Alter incentive/allowance structure)

Additional strategies included avoiding reimbursement tied to outcomes lacking clinical evidence (*use other payment schemes*) (O, OS). Physicians also emphasized the need to ensure a maximum number of patients per physician to ensure adequate time for clinical decision-making and reduce pressure to provide LVC (*use* *capitated payment*) (O, OS). Furthermore, they emphasized that practices lacking evidence should not be procured and that resources are allocated to support evidence-based alternatives (*fund and contract for clinical innovation*) (O, OS). Another suggestion was to *develop disincentives* (O), for example, by requiring physiotherapists who recommend imaging to also be responsible for the referral and associated costs, thereby discouraging unnecessary diagnostic procedures.

### Change infrastructure

Physicians suggested changes in how work is organized *(assess and redesign workflow).* Increased continuity of care, where patients consistently meet the same physician, was believed to reduce LVC by fostering trust and enabling watchful waiting (G, L). They further suggested longer appointments and fewer patients per physician to allow time for clinical reasoning and follow-up (G, L, O), as well as more patient-free time for reflection, staying updated, and education (G, L). Managers were encouraged to support deimplementation by allocating time for staff development to (*mandate change*) (L, O).

Additional proposals included changing the physical environment, such as removing bundled lab tests and introducing AI for triage (*changing physical structure and equipment)* (O, OS). Physicians also called for stricter controls on professional competence, including the licensing of EU physicians and oversight of substitute and specialist physicians (*create or change credentialing and/or licensure standards*) (O, OS). Suggestions for continuous professional development include mandatory annual training for primary care physicians (OS) and reducing reliance on temporary staff and discontinuing online care providers, which are perceived to increase LVC (*change accreditation or membership requirements*) (O, OS).

Physicians proposed reforms to the national supervisory agency and recommended that harm from overdiagnosis be assessed similarly to underdiagnosis (*changes in liability laws*) (OS). A novel strategy, *appropriate level of care* (O, OS), highlights the need for improved collaboration between primary and secondary care. It was proposed that secondary care takes greater responsibility for complex cases and avoids shifting diagnostic burdens to primary care because of limited access to better tests. Additionally, sick leave management could be transferred to occupational health or specialist units to reduce strain on primary care.

“Hospital specialists should not create new guidelines that shift tasks from hospitals to primary care/other instances, as this makes us reluctant to follow them and instead insist on referring patients.” (Appropriate level of care)

### Policy and regulations

Two new strategies related to policy and regulations were identified. Physicians highlighted the importance of nationally agreed-upon guidelines to ensure consensus on which practices should not be used (*Develop national guidelines*) (OS) and to avoid placing sole responsibility on individual physicians to enforce the deimplementation of LVC. Furthermore, there was a call to remove LVC practices from existing guidelines. Physicians referenced *choosing wisely* as a promising national initiative to support this effort. This domain also included strategies aimed at creating alignments, both across system levels and between different guidelines (*aligned* *directives across health system level*s). Information about LVC and related deimplementation efforts should be communicated through multiple channels, including governance agencies, medical education, and senior management, with clear explanations for why certain practices are removed or replaced.

“Have it decided centrally, e.g., by a committee/experts who then disseminate this to clinics where management and subsequently politicians support the decision and inform the public. Unity is key.” (Aligned directives across health system levels)

Standardized care pathways were noted to sometimes lead to unnecessary testing, and the need to assess unintended consequences of political decisions that may increase LVC use was emphasized.

### Changes in scope and nature of benefits and services

Having alternative practices to offer instead of the LVC practice was considered helpful for deimplementation. Physicians emphasize the importance of having clear information on suitable alternatives and ensuring that no barriers, such as long waiting times or high costs (O, OS), exist to their use.

“Make the new alternatives easily accessible and appealing, something people want to use when they hear about them, without long waiting times and at reasonable costs, so that they spark interest among healthcare staff. This also makes it easier to convey the motivation behind the changes. Hopefully, this will reduce the tendency to fall back on previous practices simply because they are familiar.”

### Develop better evidence

This domain has not previously been represented in the ERIC compilation or its adaptations for deimplementation. It includes requests for clearer evidence to clarify which practices are of low value. This included data beyond clinical effectiveness, such as information on costs and time required for treatment (*include cost and time in guidelines*) (O, OS), and transparency regarding the evidence base for do-not-do recommendations (*clarify evidence for recommendations*) (O, OS).

“Clearer calculation of time needed to treat (TNT) for all methods, to be able to eliminate those that provide little benefit relative to the time they take.” (Include cost and time in guidelines)

The need for guidelines to be applicable in primary care was also emphasized (*applicable guidelines*) (O, OS), which may be supported by involving general medicine specialists in guideline development. Finally, further research and development are needed for practices where the evidence base remains unclear (*R&D for practices with unclear evidence*) (G, O).

## Discussion

This study explored the strategies that primary care physicians perceive to be effective in facilitating the deimplementation of LVC in primary care, as well as the system levels responsible for deploying these strategies. Strategies have been suggested across all ERIC domains and the additional domains and strategies previously identified [13, 16, 38]. Furthermore, several novel strategies have been proposed: one categorized in the *change infrastructure* *domain*, two within the *policy* *and regulations* domain, and four within a novel domain *labeled “develop better evidence”*. The most frequently identified levels for making decisions to deploy strategies were the organizational and overarching levels.

ERIC compilation does not provide explicit guidance regarding the appropriate system level for the deployment of strategies. Research has shown that deimplementation strategies have focused primarily on individual healthcare professionals, most often through educational interventions [16]. However, primary care physicians provide numerous examples showing that factors beyond a lack of knowledge concerning LVC influence their provision of it and that they are often left to address these challenges on their own [20]. To our knowledge, deimplementation research has not previously combined a comprehensive strategy taxonomy (ERIC) with an explicit system-level mapping. By integrating these perspectives, this study moves beyond simply identifying strategies that may support de‑implementation to clarifying where in the system action is feasible. Our study reveals that most deimplementation strategies suggested by physicians cannot be initiated by them or by their PCC and thus rely on engagement from larger organizational or overarching level actors to support deimplementation efforts. The organization and overarching level might imply different things in different settings but include decision making at higher levels, reimbursement, education systems, laws, etc. Thus, this study highlights a broader set of strategies across multiple system levels, emphasizing the need to distribute responsibility for deimplementation throughout the healthcare system rather than resting solely on individual physicians.

The deimplementation strategies suggested by the physicians in our study differ from those previously evaluated in the scientific literature. For example, few strategies in the ERIC domain *develop stakeholder interrelationships* have been evaluated for deimplementation purposes [13], although they have been suggested to be particularly effective in reducing LVC [16]. The physicians in this study emphasized the relevance of a range of strategies within this domain, including *conducting local consensus discussions*, *informing local opinion leaders*, *building coalitions*, *promoting network weaving, visiting other sites, using advisory boards and workgroups,* and *involving executive boards*. Their responses highlight a demand for greater involvement and collaboration across multiple levels of the healthcare system and the need to evaluate these strategies in future studies.

Another proposed type of strategy in our study was related to the *change* *infrastructure* domain, which includes strategies such as *assess and redesign workflow* and *change physical structure and equipment*. This type of strategy has shown effectiveness, leading to consistent reductions in LVC practices in nine of 13 systematic reviews on strategies for reducing LVC [16]. Previous deimplementation efforts have employed strategies such as altering the physical structure or equipment to make it more difficult to use an LVC practice and/or easier to adopt an alternative practice. Our study, however, identified additional requests for infrastructure changes at the organizational and system levels, including modifications to credentialing and licensure standards, accreditation or membership requirements, liability laws, and ensuring that care is provided at the appropriate level.

A previous deimplementation review identified the use of mass media as the only strategy used within the *engage consumer* domain [13]. While the physicians in our study also recognized mass media as a strategy, they proposed additional strategies aimed at reducing patient-driven demand for LVC and empowering patients to take an active role in its reduction. For example, using strategies to decrease demands for LVC, supporting physicians in how to communicate with patients about refraining from a practice (*communication* *tool*) and involving patients in decisions about their care (*prepare* *patients to be active participants)*. Given that patient requests are among the most reported reasons for providing LVC [9, 33], these strategies may be particularly impactful. Accordingly, involving patients in deimplementation efforts has been strongly recommended [33, 39]. In particular, strategies centered on shared decision-making have shown promise in reducing the use of LVC [39].

Several proposed strategies in our study aligned with those specifically added to the ERIC for deimplementation purposes [13, 16]. Moreover, strategies not previously identified, neither in ERIC nor the additions, were suggested. This implies that while the ERIC compilation includes numerous strategies that might function for deimplementation, there are significant distinctions between strategies for implementing evidence and those for deimplementing LVC that merit attention. The novel deimplementation strategies within the domain *develop better evidence* may be particularly important for deimplementation, as insufficient or ambiguous evidence presents a significant barrier to the deimplementation of LVC [40, 41]. Although it is well known that providing evidence is seldom sufficient for behavior change (e.g., [42]), the results underline that the type of evidence may matter, and possibly specifically so for deimplementation recommendations. Evidence on the financial implications and time consumption of LVCs may be needed, along with clinical outcomes. A challenge for deimplementation recommendations is that the mere absence of evidence of effects is not as convincing as evidence proving that a practice is ineffective [43]. Conducting studies to provide such evidence is challenging, and since the practice is already in use, conducting randomized controlled trials is often unfeasible. Routine health system data can support research and development initiatives for established practices. However, there is a need for systems and metrics that effectively capture LVC utilization and outcomes [43]. The physicians in our study highlighted this necessity, yet current opportunities to assess LVC utilization within the Swedish healthcare system remain limited [31].

### Methodological considerations

The study has several strengths. It offers a comprehensive exploration of strategies for deimplementing LVC, drawing directly from more than 400 primary care physicians’ suggestions and covering all ERIC domains along with additional adapted deimplementation strategies. All Swedish healthcare regions were represented as well as both publicly and privately operated PCCs. The responses to the open-ended question provided rich data on potentially useful strategies. The physician-centered approach was a strength, ensuring that the recommendations align with real-world clinical practice and the challenges faced by primary care physicians. By tailoring its findings to the Swedish primary care context, the study ensures practical relevance while also providing insights that may be applicable in other healthcare systems. The methodological rigor, through qualitative analysis structured within established frameworks, enhances the reliability and interpretability of the findings.

However, several limitations should be considered. The study only includes Swedish physicians, and thus, interpretation of system levels is reflecting of the Swedish health care system. As responsibilities and mandates vary between countries, specific strategies may be most appropriately deployed on other levels in other health care systems. The sample is also limited to one professional group (physicians) and one setting (primary care), and the design does not support analysis of prevalence or representativeness.

The open-ended question focused on LVC in general and not on a specific LVC practice. This means that the physicians may have considered different types of LVC practices and that some strategies that would have been specifically appropriate for certain types of LVC were not mentioned. Different types of LVC practices may require different (combinations of) deimplementation strategies as the determinants for their use can vary [9, 13, 14]. Nevertheless, the data revealed a breadth of suggested strategies that cut across different levels that can be used to inform the choice of deimplementation strategies for specific LVC practices. It should be noted that this study focused on deimplementation strategies suggested by physicians, which do not necessarily reflect those that are most appropriate or effective in clinical practice.

Another limitation of the open-ended survey method was the inability to pose follow-up questions, which resulted in varying levels of detail in the responses. Not all the suggestions explicitly indicated the appropriate level for strategy deployment. In cases where this information was lacking, four researchers assessed and assigned the most appropriate levels using the IGLOO framework. This process involved latent interpretation, which is an inherent and methodologically accepted part of qualitative content analysis. To ensure methodological rigor, all four researchers conducted the categorization jointly and discussed each coding decision until consensus was reached. While this analytical approach provided a structured basis for categorization, it also introduced an element of interpretation that may not fully capture the informants’ original intentions. To enhance transparency and credibility, we provide detailed examples in Additional File 2 illustrating how latent interpretations were made and how these were grounded in the empirical data.

Importantly, the newly proposed domain *develop better evidence* and the *policy and regulations domain* have not been developed via concept mapping, unlike the original ERIC domains. Future research is needed to determine whether these newly proposed strategies are relevant across different healthcare settings.

## Conclusion

Swedish primary care physicians envision a range of deimplementation strategies to reduce LVC. They proposed strategies across all nine ERIC domains, with additional strategies beyond those previously described, including a domain: *develop* *better evidence*. From the physicians’ perspective, deimplementation cannot rely solely on individuals but requires coordinated action across organizational and overarching levels. Broader structural, policy, and cultural changes would support physicians and ensure that responsibility for deimplementation is shared across the healthcare system.

## Supplementary Information

Additional file 1.

Additional file 2.

## Data Availability

The datasets used and/or analyzed during the current study are available from the corresponding author upon reasonable request.

## Abbreviations

ERIC: Expert recommendations for implementing change
IGLOO: Individual, Group, Leader, Organization, Overarching/social
LVC: Low-value care
PCC: Primary Care Center
