# Supplementary material for: Physician-suggested strategies for deimplementing low-value care in Swedish primary care: a qualitative analysis across system levels
Source: Implement Sci Commun. 2026 Apr 18;7:75. doi: 10.1186/s43058-026-00947-6 (PMC13094004; doi:10.1186/s43058-026-00947-6)
Supplement: Supplementary file 2 — Additional file 2. [file 43058_2026_947_MOESM2_ESM.pdf]

Additional file 2. Example of coding based on the Expert Recommendations for Implementing Change (ERIC) compilation and the IGLOO framework

The table below provides one example of coding for each domain in the ERIC compilation [1] and its later additions [2-4] mapped onto the levels in the IGLOO framework (individual, group, leader, organization and overarching/social system) [5]. We defined the individual level as the individual physician; the group level as the primary care center (PCC); the leader level as PCC management; the organizational level as the primary care organization within a healthcare region; and the overarching/social context as the wider overarching system, encompassing primary care across multiple regions, secondary care, government agencies and national educational institutions.

For each example, we also clarify whether our interpretation was based on explicit indications of system level in the strategy suggestions (manifest interpretation), or whether it was inferred through latent interpretation grounded in participants' descriptions and further supported by our contextual knowledge of the Swedish healthcare system

**Domain**	**Discrete strategy**	**Physician suggested strategy <u>(IGLOO-level)</u>**	**Manifest/latent interpretation**
Use evaluative and iterative strategies	Audit and provide feedback	Feedback **<u>at workplace and individual level (physicians, nurses)</u> (G,L)** with a short report describing the current situation and improvement suggestions, followed by follow-up.	Manifest
		Ongoing **monitoring from the region (O)**, for example through the statistics that are presented, where my health centre is compared with other centres in the same area. If we then see that we issue 50% more referrals for colonoscopies, that will likely make us stop and reflect.	Manifest
	Provide clinical supervision	Good support from **<u>senior colleagues (I)</u>**	Manifest

Provide interactive assistance		Sufficient time allocated for debriefing immediately after the visit for trainee doctors would likely reduce overdiagnosis by allowing them to feel confident and deliberate about postponing further investigation for the moment. **(G,L)**	Latent. No specific IGLOO level was mentioned. As PCCs have autonomy to decide how they allocate time for different tasks, this was categorized as group and leader level.
Adapt and tailor to context	Tailor strategies	The choice of intervention depends on the specific situation and the underlying causes of using ineffective methods. It is probably most effective to use a combination of these strategies to achieve the best result. **(IGLOO)**	Latent. No specific IGLOO level was mentioned. As the level of deployment would be decided by the type of intervention we mapped this to all IGLOO levels.
	Promote adaptability	Reduced detailed steering in central guidelines/standardized care pathways and increased focus on qualitative measures in quality improvement work. **(OS)**	Latent. N specific IGLOO level was mentioned. Because we know that guidelines are issued at national and regional level this was categorized as Overarching/social system level.
Develop stakeholder interrelationships	Conduct local consensus discussion	More time allocated for collegial dialogue **<u>at, for example, physicians' meetings</u> (G,L),** where new findings and individual patient cases can be discussed as a group.	Manifest
		**Physicians at the same workplace (G,L)** should follow the guidelines in the same way to reduce patients going around to different colleagues… if it's a difficult patient with	Manifest

		strong demands, then it should be discussed at a physician meeting and a joint decision given to the patient.	
Train and educate stakeholders	Conduct ongoing training	Scheduled, regular collegial discussion forums with a permissive attitude, **sanctioned and encouraged by managers and leadership (G, L).** Ideally, especially in the beginning, with an external facilitator who presents data such as prescribing patterns, referral statistics, laboratory use, and so on.	Manifest
		**Discussions led by the medical director at the local workplace (L)**. Based on guidelines and facts about which investigations/treatments have a high time needed to treat, and which are common 'time thieves' or 'money drains' in our unit.	Manifest
		More continuing education for physicians to give them the opportunity to access the latest guidelines so they can be implemented in everyday care, and strengthen them in daring to say no to demanding patients. **(G, O, OS)**	Latent. No specific IGLOO level was mentioned by the respondent. At the group level, the PCC is responsible for providing opportunities for physicians to participate in continuing education, while the organizational and overarching system levels are responsible for

			ensuring the necessary prerequisites and educational resources.
Support clinicians	Remind clinicians	**<u>Linking together different guidelines, for example Viss.nu</u>** **<u>(O)</u>** [Regional guidelines] with the medical record system, so that you are alerted if you perform an action that is not recommended.	Latent. No specific IGLOO level was mentioned. Because we know that viss.nu is a knowledge support resource for those who work in primary care within a healthcare region, we mapped this to the organizational level.
		Fewer and simpler decision-support tools that also take cost-effectiveness and displacement effects into account. **(O)**	Latent. No specific IGLOO level was mentioned. Based on typical practice, decision support tools are usually designed and deployed at the organizational level.
		Digital decision support linked to the medical record system so that reminders appear in the moment. **(O)**	Latent. No specific IGLOO level was mentioned. Based on typical practice, decision support tools are usually designed and deployed at the organizational level.
Engage consumers	Decrease demand (Increase demand in the original ERIC compilation)	For patients, it often feels like healthcare wants to deny them something, and in that situation, patients do not listen well. **<u>Information from, for example, the</u>**	Manifest

		**National Board of Health and Welfare that the patient can take home (OS),** along with a promise of a new discussion, usually provides more time and space to better understand the evidence and see the pros and cons of testing or diagnostics.	
		For example, **conducting informational campaigns in public places (OS)** such as: 'Did you know that a cough caused by a common cold resolves on its own without cough medicine and is usually caused by a virus that does not require antibiotic treatment?' Today, however, we tend to hear messages emphasizing that it should be easy to contact a doctor, which I interpret as meaning that people are encouraged to seek medical care for all sorts of issues, as if everything were equally important, low value care versus complex multimorbidity.	Latent. No specific IGLOO level was mentioned. As public information campaigns would typically be the responsibility of regional or national level actors in Sweden, this was categorized as the overarching/social system level.
Utilize financial strategies	Alter incentive/allowance structures	Link reimbursement to the use of effective methods and the results they generate. **(OS)**	Latent. No specific IGLOO level was mentioned. It is the health and medical care boards or the primary care boards in each region that make political decisions about reimbursement,

			therefore we mapped this to the overarching/social level.
		Changed financial incentives (OS). I believe in Minna Johansson's arguments [...], including that poor quality indicators corrupt and lead us to do unnecessary things just to achieve good numbers. I believe this needs to change. **(OS)**	Latent. No specific IGLOO level was mentioned. It is the health and medical care boards or the primary care boards in each region that make political decisions about reimbursement, therefore we mapped this to the overarching/social level.
Change infrastructure	Appropriate care level (new strategy)	Accessible support from **secondary care (OS)**, so that we don't have to manage investigations and treatments we don't have experience with.	Manifest
		**Hospital specialists should not create new guidelines that shift tasks from hospitals to primary care/**other instances **(OS),** as this makes us reluctant to follow them and instead insist on referring patients.	Manifest
Policy and regulations [4]	Aligned directives across health system levels (new strategy)	There needs to be extraordinary clarity all the way from **the National Board of Health and Welfare and through the educational institutions across the country (OS),** so that the same message is conveyed consistently at	Manifest

		every level of training: undergraduate education, internship (AT), basic training (BT), specialist training (ST), and continuing professional development for established specialists.	
		Have it decided centrally, e.g., by a committee/experts who then disseminate this to clinics where management and subsequently politicians support the decision and inform the public. Unity is key. **(OS)**	Latent. No specific IGLOO level was mentioned explicitly but overarching/social level was indicated by the word "centrally" and targeting managers, politicians and the public.
Changes in scope and nature of benefits and services [3]	Changes in scope and nature of benefits and services	Clear, well-justified guidelines AND clear, well-justified alternatives. Otherwise, I think it could cause a diagnostic vacuum. **(O, OS)**	Latent. No specific IGLOO level was mentioned. Because guidelines are issued at both national and regional primary healthcare levels we mapped this strategy to the organization and overarching/social level.
		Make the new alternatives easily accessible and appealing, something people want to use when they hear about them, without long waiting times and at reasonable costs, so that they spark interest among	Latent. No specific IGLOO level was mentioned. We categorized this as the organizational and the overarching/social level as both can influence the

		healthcare staff. This also makes it easier to convey the motivation behind the changes. Hopefully, this will reduce the tendency to fall back on previous practices simply because they are familiar. **(O, OS)**	availability of practices.
Develop better evidence (new domain)	Include time and cost in guidelines	Evaluate guidelines according to “time needed to treat” since it is currently difficult to determine which interventions are resource-efficient in relation to the time required, time is generally our greatest limiting factor in primary care. **(OS)**	Latent. No specific IGLOO level was mentioned. Because guidelines are issued at national and regional level we mapped this strategy to the overarching/social level.
		Clearer calculation of time needed to treat (TNT) for all methods, to be able to eliminate those that provide little benefit relative to the time they take. **(OS)**	Latent. No specific IGLOO level was mentioned. Because guidelines are issued at national and regional level we mapped this strategy to the overarching/social level.

1. Powell, B.J., T.J. Waltz, M.J. Chinman, L.J. Damschroder, J.L. Smith, et al., *A refined compilation of implementation strategies: results from the Expert Recommendations for Implementing Change (ERIC) project.* Implementation Science, 2015. 10(21).
2. Perry, C.K., L.J. Damschroder, J.R. Hemler, T.T. Woodson, S.S. Ono, et al., *Specifying and comparing implementation strategies across seven large implementation interventions: a practical application of theory.* Implementation Science, 2019. 14(32).
3. Kien, C., J. Daxenbichler, V. Titscher, J. Baenziger, P. Klingenstein, et al., *Effectiveness of de-implementation of low-value healthcare practices: an overview of systematic reviews.* Implementation Science, 2024. 19(56).

4. Ingvarsson, S., H. Hasson, U. von Thiele Schwarz, P. Nilsen, B.J. Powell, et al., *Strategies for de-implementation of low-value care—a scoping review*. Implementation Science, 2022. 17(73).
5. Nielsen, K., J. Yarker, F. Munir, and U. Bültmann, *IGLOO: An integrated framework for sustainable return to work in workers with common mental disorders*. Work & Stress, 2018. 32(4): p. 400-417.
